# Trajectories of Depressive Symptoms among Multicultural Adolescents in Korea: Longitudinal Analysis Using Latent Class Growth Model

**DOI:** 10.3390/ijerph17218217

**Published:** 2020-11-06

**Authors:** Min Kyung Song, Ju Young Yoon, Eunjoo Kim

**Affiliations:** 1 Department of Nursing, College of Medicine, University of Ulsan, Ulsan 44610, Korea; mk0408@ulsan.ac.kr; 2 College of Nursing, Seoul National University, Seoul 03080, Korea; yoon26@snu.ac.kr; 3 Research Institute of Nursing Science, Seoul National University, Seoul 03080, Korea; 4 Center for Human-Caring Nurse Leaders for the Future by Brain Korea 21 (BK 21) four project, Seoul National University, Seoul 03080, Korea

**Keywords:** depression, adolescent, cultural diversity, migrants, latent class analysis, multicultural adolescents, panel survey, MAPS

## Abstract

The purpose of this study was to investigate the trajectory of depressive symptoms in multicultural adolescents using longitudinal data, and to identify predictive factors related to depressive symptoms of multicultural adolescents using latent class analysis. We used six time-point data derived from the 2012 to 2017 Multicultural Adolescents Panel Study (MAPS). Latent growth curve modeling was used to assess the overall features of depressive symptom trajectories in multicultural adolescents, and latent class growth modeling was used to determine the number and shape of trajectories. We applied multinomial logistic regression analysis to each class to explore predictive factors. We found that the overall slope of depressive symptoms in multicultural adolescents increased. Latent class analysis demonstrated three classes: (1) high-increasing class (i.e., high intercept, significantly increasing slope), (2) moderate-increasing class (i.e., moderate intercept, significantly increasing slope), and (3) low-stable class (i.e., low intercept, no significant slope). In particular, we found that the difference in the initial intercept of depressive symptoms determined the subsequent trajectory. There is a need for early screening for depressive symptoms in multicultural adolescents and preparing individual mental health care plans.

## 1. Introduction

Increasing international mobility and globalization has led to increasingly diverse communities worldwide [1]. The number of international migrations has steadily increased, reaching approximately 200 million in 2019, and this increase has accelerated in recent years [2]. The number of immigrants in South Korea (hereafter Korea) has also been increasing since the mid-1990s when economic growth commenced [3]. Immigrants in Korea at the end of 2019 comprised 4.86% of the total population, equating to 2,524,656 people [4]. This increase is primarily due to an increase in the number of migrant workers and international marriages [3]. The Korean government defines immigrants (including immigrants by marriage, immigrants by work, North Korean refugees, and ethnic Koreans) and their families as “multicultural families”, and provides supportive services, such as Korean language education, counseling, social and occupational training, and family education.

As a multicultural family is a combination of individuals of various cultural backgrounds, conflict or convergence related to cultural identity differences occurs. In addition, multicultural families have aspects that can affect an individual’s life and society as a whole, such as biculturalism, language, and adaptation. The increase in multicultural families is also coupled with the increase in multicultural children and adolescents. More than five out of every 100 newborns in Korea are multicultural children, and the long-term settlement ratio of multicultural families is increasing [5]. As a result, attention to these populations is needed because the proportion of children and adolescents from multicultural backgrounds will also increase rapidly.

Adolescence is a critical transitional period when individuals begin to explore their identities and positions in society as they grow up physically, psychologically, and socially [6]. During this period, they experience both rapid growth and developmental crises, and are more likely to be exposed to problem behaviors than in previous stages of development. Depression in adolescence is of great significance in that it has a profound effect on the quality of life throughout life. According to the World Health Organization, depression is one of the leading causes of illness and disability in adolescents [7]. Adolescent depression may present not only as depressed mood, but also as eating disorders, anxiety, refusal to attend school, poor academic performance, substance abuse, or behavioral problems [8]. In addition, it has been reported that adolescent depression is closely related to suicide—the leading cause of death in adolescence [9]. Previous studies have reported that childhood/adolescent depression is associated with high levels of adult depression, anxiety, and illicit drug use, as well as worsening health, criminal activity, and social functioning [10,11].

Previous studies of the correlations or trajectories of depressive symptoms among non-multicultural adolescents have considered individual characteristics, such as gender, economic status, academic achievement, body satisfaction, and self-esteem, and environmental factors, including family support, friendship, peer group support, and school and community support [12,13,14,15]. In a study involving multicultural adolescents, the following were additionally assessed: cultural adaptation stress, self-esteem, and Korean language proficiency [16]. Although studies of the factors that influence depressive symptoms among multicultural adolescents have been conducted, most studies were cross-sectional in design [17,18]. Despite recent increased availability of longitudinal data, such as panel data, there are too few extant studies to identify changes over time and factors that influence depressive symptoms among multicultural adolescents [16]. In addition, sub-groups of multicultural adolescents with distinct characteristics may exist. Unlike extant variable-centered analysis, one might differentiate latent classes with different development trajectories, namely the population might be divided into different classes according to individual response patterns (person-centered analysis) [19]. It is important to conduct basic research to clarify the predictors of depressive symptoms to determine the implications for prevention strategies and the specific support measures that are needed to address depressive symptoms in multicultural adolescents.

The purpose of this study was to investigate trajectories of depressive symptoms in multicultural adolescents using Multicultural Adolescents Panel Study data. In addition, we intended to provide a theoretical basis for interventions to alleviate depressive symptoms in future multicultural adolescents by identifying latent classes with different developmental trajectories of depressive symptoms and factors that predict class membership. The specific objectives of the study were as follows: (1) to identify the changes in depressive symptoms over time in multicultural adolescents. (2) To identify the trajectories of depressive symptoms in multicultural adolescents. (3) To investigate factors that predict classes of depressive symptoms in multicultural adolescents.

## 2. Materials and Methods

### 2.1. Data Set and Sample

We used data from the 2012 to 2017 Multicultural Adolescents Panel Study (MAPS). The MAPS consists of panel data tracked annually since 2011 and the study population consists of multicultural students enrolled in the 4th grade of elementary school in Korea. As of 2011, 1635 students or 35% of all Korean multicultural students were paneled. The original sample retention rate was 91.7% in 2012, 88.2% in 2013, 84.3% in 2014, 82.3% in 2015, 81.2% in 2016, and 77.0% in 2017. Further information on the MAPS is provided at https://www.nypi.re.kr/archive.

The outcome variable of this study was depressive symptoms. This has been investigated in the MAPS since 2012; accordingly, only data from 2012 until 2017 were used for analysis in this study. Out of the total of 1500 individuals surveyed in 2012, 1432 respondents were included in the analysis by selecting those who responded at least twice from the 2012 survey to the 2017 survey and excluding four respondents whose parents were both immigrants.

After submitting the research plan to the National Youth Policy Institute, we were provided with data that excluded personal identifiers. This study was conducted with exemption from research review by the Ulsan University Institutional Review Board (IRB no. 2020R0011-001).

### 2.2. Measures

#### 2.2.1. Outcome Variable: Depressive Symptoms

Depressive symptoms were measured using the Symptom Checklist-90-Revision (SCL-90-R) developed by Derogatis et al. [20] and standardized by Kim et al. [21]. In the MAPS, the same 10 of the 13 items in the SCL-90-R [22] were used as in the Korean Children and Youth Panel Survey (CYPS). Each item was rated on a four-point scale ranging from 1 (not at all) to 4 (extremely). The higher the total score, the greater the depressive symptoms. Han and Kahng [13] reported a Cronbach’s alpha value of 0.90. Cronbach’s alpha in the present study was 0.90 in 2012, 0.92 in 2013, 0.92 in 2014, 0.91 in 2015, 0.91 in 2016, and 0.91 in 2017.

#### 2.2.2. Predictive Factors

Individual characteristics consisted of gender, age, geographic location, obesity, body satisfaction, academic achievement satisfaction, self-esteem, and ego-resiliency. Geographical locations used data surveyed by metropolis, urban (city except metropolis), and rural areas in the MAPS. Monthly household income was surveyed in units of KRW 10,000 and was converted into log values for multinomial logistic regression analysis. Height and weight were investigated to check the growth and development of multicultural adolescents in the MAPS. The criteria for obesity were defined as a BMI (body mass index) ≥ 95th percentile for age and sex according to the 2017 Korean National Growth Charts for children and adolescents [23]. Body satisfaction was measured using the self-concept inventory developed by Han [24]. The instrument is composed of 7 sub-factors, 6 out of the 14 questions on the body self-concept factor were modified and used in the MAPS. Each item was rated on a four-point scale ranging from 1 (not at all) to 4 (extremely), two of the six items were inversely scored. The higher the total score, the higher the level of body satisfaction. Han [24] reported a Cronbach’s alpha value of 0.69. Cronbach’s alpha in the present study was 0.74. Academic achievement satisfaction was surveyed via a 4 point Likert scale (1 = not at all satisfied to 4 = very satisfied), which was used to answer the question “How satisfied are you with your grades?” Self-esteem was measured using the Coopersmith self-esteem inventory (SEI) developed by Coopersmith [25] and modified by Park and Oh [26]. The Korean version of the SEI is composed of 3 sub-factors, 4 out of 16 questions on the self-esteem factor were modified and used in the MAPS. Each item was rated on a four-point scale ranging from 1 (not at all) to 4 (extremely), and the mean score was used for analysis. Han and Kahng [13] reported a Cronbach’s alpha value of 0.83. Cronbach’s alpha in the present study was 0.79. The ego-resiliency scale in the MAPS used the same 14 items as in the CYPS. The scale was developed by Block and Kremen [27], translated by Yoo and Shim [28], and modified by Kwon [29]. Each item was rated on a four-point scale ranging from 1 (not at all) to 4 (extremely), and the mean score was used for analysis. Kwon [29] reported a Cronbach’s alpha value of 0.74. Cronbach’s alpha in the present study was 0.91.

Multicultural characteristics included the type of multicultural family, country of immigrant parent, immigrant parents’ Korean proficiency, multicultural adolescents’ Korean proficiency, and acculturation stress. The type of multicultural family was classified as immigrant father or immigrant mother. The country of parents surveyed was classified as Korea, China (Han Chinese and other ethnic groups), China (Korean-Chinese), Vietnam, the Philippines, Thailand, Japan, and other. In this study, immigrant parents were classified as Chinese (Han Chinese and other ethnic groups, Korean-Chinese), Southeast Asian (Vietnam, the Philippines, Thailand), Japan, and other. Korean proficiency was measured on a 4 point Likert scale (1 = poor, 2 = fair, 3 = good, 4 = very good) in four categories: speaking, writing, reading, and listening. The mean score was used for the analysis. Cronbach’s alpha for immigrant fathers, immigrant mothers, and multicultural adolescents’ Korean proficiency was 0.94, 0.89, and 0.94, respectively. Acculturative stress refers to the psychological pain experienced by individuals or groups when adapting to another culture [30,31]. The level of acculturative stress was measured with a short version of the Social, Attitudinal, Familial, and Environmental (SAFE) scale developed by Hovey and King [30] and modified by Hong [31]. The scale consists of 10 items (e.g., “Because my parents are immigrants, I am ignored.”), and each item is rated on a four-point scale ranging from 1 (not at all) to 4 (extremely). The final item is inverse scored and the total score was used for analysis. Hong [31] reported a Cronbach’s alpha value of 0.71. Cronbach’s alpha in the present study was 0.76.

Environmental characteristics consisted of family support, parenting attitude (neglect), peer support, bullying experience (as a victim), teacher support, and school adaptation (learning). Support from family, peers, and teachers was assessed using the Social Support Appraisal Scale of Han [32]. The Social Support Appraisal Scale is composed of 8 items for each factor, but after a pilot test, the MAPS utilized 7 items for family support, 7 items for peer support, and 6 items for teacher support, after excluding questions with low factor loadings. In addition, in the original version, all responses were obtained via a 5 point Likert scale (1 = not at all to 5 = extremely), but the MAPS modified the family support item to a 4 point Likert scale (1 = not at all to 4 = extremely). The mean score was used for the analysis. In Han [16], Cronbach’s alpha was 0.95 for family support, 0.96 for peer support, and 0.95 for teacher support. In this study, the respective values were 0.94, 0.96, and 0.95. Parenting attitude (neglect) was measured using the same seven items as in CYPS. Each item was rated on a four-point scale ranging from 1 (not at all) to 4 (extremely) and the mean score was used for analysis. Two of the seven items were inverse scored. The mean score was used for the analysis. Lee and Choi [33] reported a Cronbach’s alpha value of 0.81 and the value in the present study was 0.77. Experience of being a victim of bullying was assessed in the MAPS using six items developed by Lee and Rhee [34]. Each item was rated on a four-point scale (1 = never, 2 = once or twice a month, 3 = once or twice a week, 4 = almost every day) and the mean score was used for analysis. Lee and Rhee [34] reported a Cronbach’s alpha value of 0.88, while the value in the present study was 0.90. School adaptation (learning) was measured using the same five items as in the CYPS. Each item was rated on a four-point scale ranging from 1 (not at all) to 4 (extremely). The final item was inverse scored, and the total score was used for analysis. Eun et al. [35] reported a Cronbach’s alpha value of 0.77. Cronbach’s alpha in the present study was 0.72.

### 2.3. Data Analysis

The characteristics of multicultural adolescents were confirmed using descriptive statistics. Latent growth curve modeling (LGCM) was performed to examine the trajectory of depressive symptoms in multicultural adolescents using data from 2012 to 2017. The initial intercept and slope of the depressive trajectory of all multicultural adolescents were confirmed by an unconditional model analysis without covariates. Goodness of fit was assessed by calculating and comparing chi-square values, comparative fit index (CFI), Tucker–Lewis index (TLI), and root mean square error of approximation (RMSEA). Latent class growth modeling (LCGM) was used to classify the groups (i.e., latent classes) according to the depressive symptom trajectory of multicultural adolescents. The analysis used Akaike information criterion (AIC), Bayesian information criterion (BIC), adjusted BIC, entropy, and the Lo–Mendell–Rubin likelihood ratio test to determine the number of latent classes. Missing data in class indicators were addressed using full-information maximum likelihood (FIML). After identifying latent classes with different trajectories of depressive symptoms in multicultural adolescents, a multinomial logistic regression analysis was performed to identify predictors of classes. IBM SPSS v.23 (IBM, Armonk, NY, USA) was used for descriptive statistics and multinomial logistic regression analysis, and Mplus 8.4 (Muthén & Muthén, Los Angeles, CA, USA) was used for the LGCM and the LCGM.

## 3. Results

### 3.1. Multicultural Adolescents’ Characteristics and Changes in Depressive Symptoms

The general characteristics of the multicultural adolescents in the 2012 MAPS data are shown in Table 1. Of the total respondents, 48.9% (n = 700) were boys and 51.1% (n = 732) were girls. The mean age was 10.97 ± 0.36 years, and most respondents were 11 years old (n = 1267, 88.5%). A total of 379 multicultural adolescents (26.5%) lived in a metropolis, 650 adolescents (45.4%) lived in urban areas, and 403 adolescents (28.1%) lived in rural areas. The mean monthly household income was approximately KRW 2.18 million. In total, 66.3% of multicultural adolescents (n = 950) were obese. The average body satisfaction score was 17.69 ± 3.01. The average satisfaction with academic achievement was 2.78 ± 0.77, that for self-esteem was 3.17 ± 0.55, and that for ego-resiliency was 2.95 ± 0.47.

Among the multicultural families, 1376 multicultural adolescents (96.1%) had an immigrant mother, and 56 multicultural adolescents (3.9%) had an immigrant father. Of the former, the greatest proportion of mothers originated from Japan and other countries, followed by Southeast Asia and China; of the latter, most fathers also originated from Japan and other countries, followed by Southeast Asia and China. The average Korean proficiency of immigrant parents was 3.14 ± 0.57, while the average Korean proficiency of the multicultural adolescents was 3.63 ± 0.50. The average acculturation stress level of multicultural adolescents was 14.43 ± 3.66.

The average family support score was 3.18 ± 0.59, and the average neglected parenting attitude was 1.85 ± 0.55. The average peer support score was 3.89 ± 0.84, and the average bullying experience score was 1.12 ± 0.35. The average teacher support score was 3.66 ± 0.89, and the average school adaptation (learning) score was 2.93 ± 0.49.

The mean depressive symptom scores over time were 16.02 ± 5.26 in 2012, 16.08 ± 5.27 in 2013, 16.40 ± 5.34 in 2014, 16.94 ± 5.36 in 2015, 17.15 ± 5.36 in 2016, and 17.40 ± 5.52 in 2017.

### 3.2. Trajectory of Depressive Symptoms among Multicultural Adolescents

Table 2 shows the trajectory of depressive symptoms among multicultural adolescents from 2012 to 2017. The model provided good fit to the data (CFI = 0.964, TLI = 0.966, and RMSEA = 0.052). The depressive symptom trajectory of multicultural adolescents was statistically significant with an initial average intercept of 15.878 (*p* < 0.001) and average slope of 0.330 (*p* < 0.001).

### 3.3. Latent Class According to the Trajectory of Depressive Symptoms of Multicultural Adolescents

Table 3 presents AIC, BIC, adjusted BIC, entropy, Lo–Mendell–Rubin (LMR) likelihood ratio tests, and the numbers and proportion of individuals in each class of each latent class growth model. Although AIC, BIC, and adjusted BIC values continued to diminish, the LMR test failed to reach significance from the four-class model onwards. Hence, the three-class model provided the best fit to the observed data. When the latent classes were divided into three, the proportions of individuals within each class was 8.9% (n = 128) in class one, 46.9% (n = 672) in class two, and 44.1% (n = 632) in class three.

**Figure 1:**
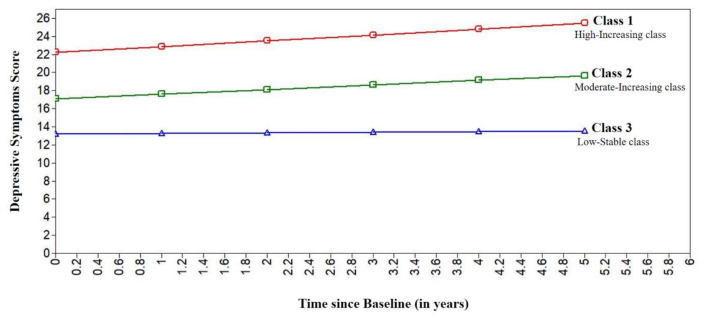
Latent classes of depressive symptom trajectories.

Table 4 and Figure 1 show the course trajectories of the three latent classes identified. The first class, which was named “high-increasing class”, was characterized by high initial depressive symptoms (intercept = 22.218, *p* < 0.001) that subsequently increased (slope = 0.644, *p* < 0.001). The second class, which was named “moderate-increasing class”, began with depressive symptom values between the first and third classes (intercept = 17.073, *p* < 0.001) and exhibited a relatively modest increase compared to the first class (slope = 0.514, *p* < 0.001). The third class, which was named “low-stable class”, began with the lowest depressive symptoms score of the three classes (intercept = 17.073, *p* < 0.001) and maintained this level over time (slope = 0.063, *p* = 0.292).

### 3.4. Predictors of Identified Course Trajectories

The Durbin–Watson test indicated that there was no autocorrelation within the residuals (Durbin–Watson statistic = 1.901). There was no multicollinearity between independent variables, with tolerance of 0.469–0.968 and a variance inflation factor of 1.033–2.133.

In multinomial logistic regression analysis, gender and geographic location were statistically significant predictors of latent class membership (Table 5). Compared to the reference (class two: moderate-increasing class), boys were less likely to belong to class one (high-increasing class, odds ratio (OR) = 0.435, 95% CI = 0.281–0.674) and more likely to belong to class three (low-stable class, OR = 2.066, 95% CI = 1.617–2.642). Multicultural adolescents living in urban areas were more likely than those in rural areas to belong to the high-increasing class rather than the moderate-increasing class (OR = 1.725, 95% CI = 1.036–2.872). In contrast, multicultural adolescents living in urban areas were less likely than those in rural areas to belong to the low-stable class rather than the moderate-increasing class (OR = 0.600, 95% CI = 0.448–0.804). A low-stable class rather than a moderate-increasing class was predicted by greater ego-resiliency (OR = 1.518, 95% CI = 1.047–2.201) and lower acculturation stress (OR = 0.944, 95% CI = 0.909–0.981).

Compared to the moderate-increasing class, high family support predicted a low-stable class (OR = 1.369, 95% CI = 1.058–1.772) and a highly neglectful parenting attitude predicted a high-increasing class (OR = 1.656, 95% CI = 1.085–2.529). Regarding school life, low peer support predicted a high-increasing class (OR = 0.714, 95% CI = 0.521–0.977). In addition, greater experience of being a victim of bullying was associated with a high-increasing class (OR = 1.651, 95% CI = 1.096–2.489), and less experience of being a victim of bullying was associated with a low-stable class (OR = 0.491, 95% CI = 0.293–0.825). The greater the level of school adaptation, the more likely adolescents were to belong to the low-stable class rather than the moderate-increasing class (OR = 1.715, 95% CI = 1.216–2.419).

## 4. Discussion

The current study used a latent class growth model to investigate the trajectory of changes in depressive symptoms according to the growth of multicultural adolescents, with the secondary aim of identifying risk and protective factors for this group.

On average, the depressive symptom of multicultural adolescents increased linearly over time, which is a similar finding to a recent study of multicultural adolescents [16]. In the current study, the trajectory of depressive symptoms identified by the latent growth curve model started at 15.878 points in the first year (fifth grade) and increased by 0.33 points every year for 6 years. In addition, this is consistent with a prior study [2] that confirmed the developmental trajectory of depressive symptoms from the fourth through to eighth grades using longitudinal data from the Korean Youth Panel Study (KYPS) [15]. Differences in the initial value of depressive symptoms are factors related to the subsequent slope. Considering the results of previous studies, which have found that children’s depressive symptoms affect them even into adulthood [32], early detection of multicultural children and adolescents who are vulnerable to depressive symptoms and providing interventions are of paramount importance, and thus early screening is necessary.

Given the heterogeneity of longitudinal trajectories of depressive symptoms over time, the present study classified individual trajectories among multicultural adolescents into three latent classes: “High-Increasing class”, “Moderate-Increasing class”, and “Low-Stable class.” The high-increasing class exhibited the highest level of depressive symptoms among classes in the fifth grade. The level of depressive symptoms in this class continued to increase subsequently and this class exhibited the highest level of depressive symptoms among classes in the 10th grade. This class accounted for 8.9% of the total sample (n = 128). The moderate-increasing class reported slightly higher depressive symptoms than the average in the fifth grade, which continuously increased to a high level of depressive symptoms in the 10th grade. This class accounted for 46.9% of the total sample (n = 672). Finally, the low-stable class exhibited a consistently lower than average level of depressive symptoms than the average from fifth to 10th grade, 44.1% (n = 632) of the total study subjects belonged to this class. The three classes of latent trajectories identified in the current study are consistent with previous studies of depressive symptoms in children and adolescents conducted in other countries [36]. A previous systematic review [36] identified symptom trajectories of “No or low,” “Moderate”, “Increasing and high”, and “Decreasing”, of which “No or low” was the most common at 67%. In the current study, the moderate-increasing class was the most common at 46.9%, and a decreasing trajectory was not identified. Gender, parent, and peer support, which affected the classification of depressive symptom trajectories in multicultural adolescents, were also identified as risk/protective factors in a previous review for adolescent depressive symptoms [36].

In the current study, factors that predicted the developmental trajectory of depressive symptoms in multicultural adolescents were explored by dividing them into individual factors, multicultural factors, and environmental factors. Female multicultural adolescents were most likely to belong to the high-increasing class, and boys were most likely to belong to the low-stable class. These findings are consistent with the findings of studies related to depressive symptom trajectories in adolescents, namely that there is a gender difference in adolescent depressive symptom levels [36,37]. In addition, cross-sectional and longitudinal studies of multicultural adolescents have also shown that female adolescents typically report a higher level of depressive symptoms than males do [14,16]. This highlights the need for differentiated interventions for female multicultural adolescents. In addition, lower ego-resiliency was associated with higher likelihood of belonging to the low-stable class versus the moderate-increasing class. Ego-resiliency enables individuals to positively accept themselves, other people, and heterogeneous cultures, and to overcome cultural conflicts and confusion [38]. Therefore, it is necessary to support programs that improve the ego-resiliency of multicultural adolescents.

Among multicultural factors, the lower the acculturation stress, the higher the likelihood of belonging to the low-stable class compared to the moderate-increasing class. The Korean language proficiency of immigrant parents or adolescents was not statistically significant as a predictor for distinguishing between the moderate-increasing class and low-stable class. These findings are consistent with previous research that has reported acculturation stress is a risk factor for depressive symptoms and mental health issues in multicultural adolescents [13,16,39]. Children of multicultural families experience acculturation, which includes differences in parental values and attitudes towards life caused by parents’ bicultural background [16]. Children are vulnerable while growing up, often experiencing mental pain due to social prejudice or bullying, and stresses such as alienation and confusion about identity [40]. Acculturation stress, which is a representative behavioral response that multicultural adolescents experience during cultural adaptation [41], may be an additional stress factor that non-multicultural adolescents do not experience. Multicultural adolescents in Korea are exposed to bicultural and bilingual environments. According to a previous study, even multicultural adolescents born in Korea experience a sense of disparity among their peers, become subject to discrimination, and suffer serious identity confusion due to differences in their appearance or Korean proficiency [42]. In particular, multicultural adolescents are more likely to experience acculturation stress in the context of a racially homogeneous society such as Korea versus more diverse societies [43]. According to the results of this study, if acculturation stress is low, the probability of belonging to the low-stable class is high. Therefore, it is necessary for multicultural adolescents to receive screening for acculturation stress and support to alleviate such stress.

Regarding environmental factors, depressive symptoms among multicultural adolescents were more likely to occur in the high-increasing class compared to the moderate-increasing class with less peer support or more experience of being a victim of bullying. In addition, the less bullying multicultural adolescents experience, the more likely they were to be in the low-stable class compared to the moderate-increasing class. In adolescence, the desire to be independent from family becomes stronger, and the peer group becomes the center of important social relations. By becoming more immersed in the peer group, the individual can maintain an equal relationship with peers [44]. Therefore, multicultural adolescents would benefit from additional attention and support from families, schools, and local communities so that such adolescents can form positive peer relationships. Programs, such as multicultural mentoring programs, need to provide opportunities for individuals to form diverse relationships. These approaches would help to reduce school violence and improve adjustment to the school environment. In addition, there is a need to strengthen education on the prevention of violence to prevent children from multicultural families from being victims of school violence. Furthermore, it is necessary to identify the various characteristics of multicultural adolescents who experience school-based violence and develop school violence prevention programs that recognize these characteristics.

The higher the level of family support, the greater the likelihood of belonging to the low-stable class compared to the moderate-increasing class. The higher the parental neglect, the greater the likelihood of belonging to the high-increasing class compared to the moderate-increasing class. These results are consistent with studies showing that the greater the family support or the better the relationship between parents, the lower the depressive symptoms of adolescents. Studies have also reported that abuse and poor family relationships are related to depressive symptoms in adolescents [12,45]. Therefore, to lower the level of depressive symptoms in multicultural adolescents, interventions that increase the level of family support are required, including education for parents regarding appropriate attitudes. In addition, the higher the level of school adaptation (learning), the more likely the individual was to belong to the low-stable class rather than the moderate-increasing class. School is very important for adolescents because it is a place where they spend as much time as in their home and where most of their social life experiences occur. Achievement in school life is closely related to future social adaptation. The characteristics of school adaptation that multicultural adolescents exhibit occur, in part, because they are members of multicultural families, but also as part of the process of development, similar to non-multicultural adolescents. Therefore, it is necessary to provide services according to the developmental characteristics and needs of individual adolescents, while considering integration with adolescents in general.

In this study, it was found that latent class membership was not significantly associated with monthly household income, immigrant parents’ country of origin, Korean proficiency of immigrant parents and multicultural adolescents, and self-esteem. Most of the multicultural adolescents included in these panel data are children of international married families. These multicultural adolescents have lived in Korea since birth and have been around for more than 10 years, so the parents’ and adolescents’ Korean proficiency may not be considered to be a major issue for these children. This current research found that the support system of families, peer groups, and schools has a greater impact on depressive symptoms of multicultural adolescents than multicultural characteristics such as the country of origin of migrant parents, migrant parents, and multicultural adolescents’ Korean ability. The effects of these variables tend to be similar to results previously reported for the trajectories of depressive symptoms and their predictors in general adolescents [36].

The current research identified various trajectories of depressive symptoms among multicultural adolescents and factors associated with these trajectories. Classifying the trajectories of multicultural adolescents is meaningful as an initial step to provide a theoretical basis, reliable evaluation, and preventive investment to reduce high-risk trajectories [36]. Multicultural adolescents who are likely to follow the high-risk trajectory (e.g., a girl at an urban school who experiences neglect and bullying, and does not have peer support) already have a high initial level of depressive symptoms and are more likely to subsequently develop further depressive symptoms. Therefore, early interventions are needed to detect and prevent the deterioration of multicultural adolescents who are likely to follow these high-risk trajectories.

This study has several limitations. First, since only the trajectory of the initial predictive factors was examined in this study, it is necessary to examine the effect on the trajectory of changes of the predictive factors over time (e.g., if bullying newly occurs). Second, this study only included obesity as a marker of physical status due to the limitations of the secondary data. It is necessary to include additional physical health variables that could affect depressive symptoms among multicultural adolescents. Third, since the panel itself includes only multicultural adolescents, it is impossible to determine the trajectory of depressive symptoms of non-multicultural adolescents in this study. Therefore, further research using other panel data (Korean Children and Youth Panel Survey, Panel Study on Korean Children, etc.) is needed to compare the trajectories of depressive symptoms between multicultural adolescents and monocultural adolescents.

Nevertheless, this study is of value in that it identifies different trajectories of changes in depressive symptoms among multicultural adolescents. These results illustrate the need to improve depressive symptoms among multicultural adolescents in Korea via individualized support based on the characteristics of multicultural adolescents, rather than uniform support for all adolescents. It may be important to screen for depressive symptoms in multicultural adolescents, select those who need more active intervention, and implement early interventions. In particular, the characteristics of adolescents regarding peer relationships and being the victim of bullying have considerable influence on depressive symptoms; and therefore, interventions are needed in such cases.

## 5. Conclusions

This study examined changes in depressive symptoms of multicultural adolescents for 6 years using data from the 2012–2017 Multicultural Adolescents Panel Study (MAPS) to classify latent classes with different trajectories of depressive symptoms, and to classify factors that predict latent class membership. This study confirmed that the depressive symptoms of multicultural adolescents significantly worsened over time. In addition, three latent classes were identified with different trajectories according to the changing patterns of depressive symptoms (“High-increasing class”, “Low-stable class”, and “Moderate-increasing class”). Among factors that predicted latent class membership, the greater the initial depressive symptoms, the greater the probability of belonging to the high-increasing class. Therefore, there is a need to screen for depressive symptoms in multicultural adolescents in the early stages and to prepare individualized mental health care plans. In addition, it is necessary to develop intervention strategies to alleviate depressive symptoms in multicultural adolescents by considering the characteristics of each latent class of multicultural adolescents identified in this study.

## Figures and Tables

**Table 1:** General characteristics of multicultural adolescents according to the 2012 Multicultural Adolescents Panel Study (MAPS) data (n = 1432).

Variables	Categories	n (%) or Mean ± SD	Observed Range	Possible Range
**Individual characteristics**				
Gender	Boy	700 (48.9)		
	Girl	732 (51.1)		
Age (years)		10.97 ± 0.36		
	10	105 (7.3)		
	11	1267 (88.5)		
	12	53 (3.7)		
	13	6 (0.4)		
	14	1 (0.1)		
Geographic location	Metropolis	379 (26.5)		
	Urban ^1^	650 (45.4)		
	Rural	403 (28.1)		
Monthly Household income (10,000 KRW; n = 1428)		218.92 ± 106.45	0–1000	
Obesity	Yes	950 (66.3)		
	No	482 (33.7)		
Body satisfaction		17.69 ± 3.01	7–24	6-24
Academic achievement satisfaction		2.78 ± 0.77	1–4	1–4
Self-esteem		3.17 ± 0.55	1–4	1–4
Ego-resiliency		2.95 ± 0.47	1–4	1–4
**Multicultural characteristics**				
Type of multicultural family	Immigrant father	56 (3.9)		
	Immigrant mother	1376 (96.1)		
Country of mother	Korea	56 (3.9)		
	China	373 (26.0)		
	South-East Asia	447 (31.2)		
	Japan and others ^2^	556 (38.8)		
Country of father (n = 1357)	Korea	1304 (96.1)		
	China	3 (0.2)		
	South-East Asia	6 (0.4)		
	Japan and others ^3^	44 (3.2)		
Immigrant parents’ Korean proficiency		3.14 ± 0.57	1–4	1–4
Multicultural adolescents’ Korean proficiency		3.63 ± 0.50	1.5–4	1–4
Acculturation stress		14.43 ± 3.66	10–34	10-40
**Environmental characteristics**				
Family support		3.18 ± 0.59	1–4	1-4
Parenting attitude (neglect)		1.85 ± 0.55	1–3.43	1-4
Peer support		3.89 ± 0.84	1–5	1–5
Bullying experience (victim)		1.12 ± 0.35	1–4	1–4
Teacher support		3.66 ± 0.89	1–5	1–5
School adaptation (learning)		2.93 ± 0.49	1–4	1–4
**Outcome**				
Depressive symptoms	2012	16.02 ± 5.26	10–40	10–40
	2013 (n = 1423)	16.08 ± 5.27	10–40	
	2014 (n = 1361)	16.40 ± 5.34	10–37	
	2015 (n = 1327)	16.94 ± 5.36	10–40	
	2016 (n = 1309)	17.15 ± 5.36	10–40	
	2017 (n = 1241)	17.40 ± 5.52	10–38	

^1^ city except metropolis; ^2^ Japan = 484, others = 72; ^3^ Japan = 18, others = 26.

**Table 2:** Trajectory of depressive symptoms among multicultural adolescents (n = 1432).

	Mean	SE	Model Fit			
			*x*^2^ (*p*)	CFI	TLI	RMSEA
Intercept	15.878 *	0.120	77.767 (<0.001)	0.964	0.966	0.052
Slope	0.330 *	0.032				

CFI = comparative fit index; TLI = Tucker–Lewis index; RMSEA = root mean square error of approximation; * *p* < 0.001.

**Table 3:** Determining the number of latent classes according to the development trajectory of depressive symptoms (n = 1432).

			2-Class Model	3-Class Model	4-Class Model	5-Class Model
Model fit	AIC		48,490.447	48,128.987	48,037.590	47,948.608
	BIC		48,548.382	48,202.723	48,127.126	48,053.945
	Adjusted BIC		48,513.439	48,158.250	48,073.123	47,990.412
	Entropy		0.712	0.756	0.713	0.695
	LMR test	2LL	1632.370	367.459	97.397	94.982
		*p*	<0.001	<0.001	0.218	0.129
n (%) by latent class	Class 1		577 (40.3)	128 (8.9)	589 (41.1)	117 (8.2)
	Class 2		855 (59.7)	672 (46.9)	33 (2.3)	502 (35.1)
	Class 3			632 (44.1)	508 (35.5)	304 (21.2)
	Class 4				302 (21.1)	33 (2.3)
	Class 5					476 (33.2)

AIC = Akaike information criterion; BIC = Bayesian information criterion; LMR = Lo–Mendell–Rubin; LL = log likelihood.

**Table 4:** Intercept and slope of each class.

Classes	Intercept (*p*)	Slope (*p*)
Class 1 (High-Increasing class)	22.218 (<0.001)	0.644 (<0.001)
Class 2 (Moderate-Increasing class)	17.073 (<0.001)	0.514 (<0.001)
Class 3 (Low-Stable class)	13.208 (<0.001)	0.063 (0.292)

**Table 5:** Multinomial logistic regression analysis.

Variables		Class 1 (n = 128): High-Increasing Class		Class 3 (n = 632): Low-Stable Class	
		OR	95% CI	OR	95% CI
**Individual characteristics**					
Gender	Boy	**0.435**	**0.281–0.674**	**2.066**	**1.617–2.642**
	Girl	Reference			
Age (years)		0.917	0.551–1.526	0.892	0.636–1.250
Geographic location	Metropolis	0.959	0.519–1.773	0.785	0.564–1.092
	Urban^1^	**1.725**	**1.036–2.872**	**0.600**	**0.448–0.804**
	Rural	Reference			
Monthly household income (log)		1.123	0.428–2.946	1.312	0.741–2.322
Obesity	Yes	0.959	0.618–1.489	1.115	0.862–1.441
	No	Reference			
Body satisfaction		0.973	0.895–1.058	1.001	0.951–1.055
Academic achievement satisfaction		0.933	0.696–1.251	1.017	0.854–1.211
Self-esteem		0.963	0.578–1.606	1.342	0.979–1.840
Ego-resiliency		1.349	0.744–2.443	**1.518**	**1.047–2.201**
**Multicultural** **characteristics**					
Type of multicultural family	Immigrant father	1.832	0.676–4.964	1.140	0.594–2.186
	Immigrant mother	Reference			
Country of immigrant parent	China	1.171	0.691–1.986	1.199	0.874–1.644
	South-East	0.987	0.600–1.623	1.286	0.956–1.729
	Japan and others	Reference			
Immigrant parent’s Korean proficiency		0.926	0.613–1.401	0.862	0.675–1.100
Multicultural adolescents’ Korean proficiency		1.023	0.691–1.515	1.280	0.984–1.664
Acculturation stress		1.029	0.976–1.085	**0.944**	**0.909–0.981**
**Environmental characteristics**					
Family support		0.811	0.551–1.196	**1.369**	**1.058–1.772**
Parenting attitude (neglect)		**1.656**	**1.085–2.529**	0.971	0.761–1.237
Peer support		**0.714**	**0.521–0.977**	1.057	0.859–1.300
Victim of bullying		**1.651**	**1.096–2.489**	**0.491**	**0.293–0.825**
Teacher support		1.251	0.938–1.669	1.073	0.898–1.281
School adaptation (learning)		0.695	0.404–1.198	**1.715**	**1.216–2.419**

Reference group = class 2: moderate-increasing class (n = 672); OR = odds ratio; CI = confidence interval; bold figures indicate significant associations (*p* < 0.05). ^1^ city except metropolis.
